# Author Correction: Braiding properties of worldline configurations in hardcore lattice bosons

**DOI:** 10.1038/s41598-023-37786-7

**Published:** 2023-07-07

**Authors:** Fabio Lingua, Wei Wang, Liana Shpani, Barbara Capogrosso-Sansone

**Affiliations:** 1 Department of Physics, Clark University, Worcester, Massachusetts 01610 USA; 2 Department of Physics and Astronomy, Dartmouth College, Hanover, New Hampshire 03755 USA; 3 Tsung-Dao Lee Institute, Shanghai Jiao Tong University, Shanghai, 201210 China

Correction to: *Scientific Reports* 10.1038/s41598-022-22894-7, published online 29 October 2022

The original version of this Article contained an error in Figure 3 where the dataset for the VBS case was incorrect. As a result, the VBS column was updated and the last four rows of column “Z_2 n=1/3” were removed. The original Figure 3 and accompanying legend appear below.

**Figure 3:**
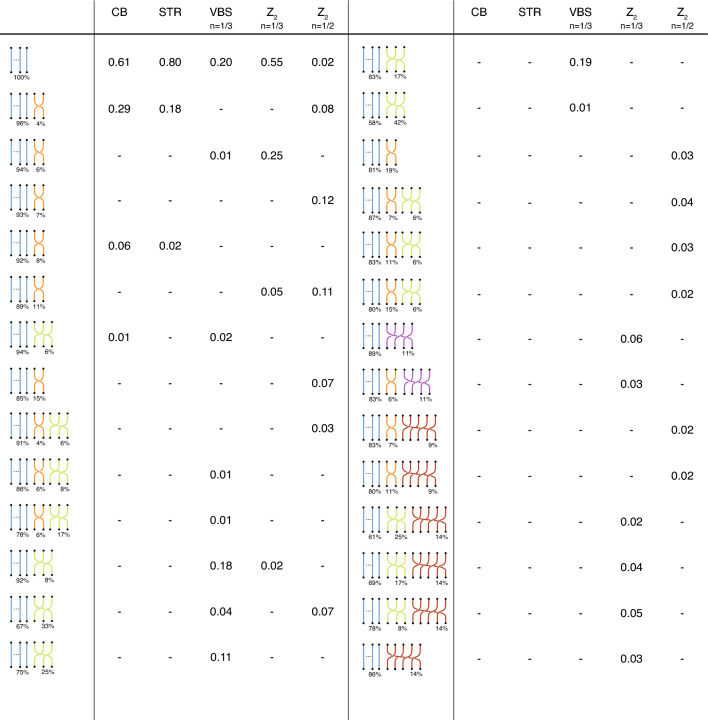
Arrangement of permutation cycles in ground-states of different insulating quantum phases. Numbers in the table represents the probability of each arrangement to appear in the related phase. The percentage in the diagrams refers to the components of $\vec {p}_\phi$ (i.e. the fraction of particles participating in permutation cycles of that length). CB for $L=10$ and $V/t=20$; STR for $L=12$ and $V/t=20$; VBS at $n=1/3$ for $n=1/3$ for $L=6$ unit cells and $V/t=30$; ${\mathbb {Z}}_2$ at $n=1/3$ and $n=1/2$ for $L=6$ unit cells and $V/t=15$.

In addition, in the Numerical results,

“In the VBS case, 57% of configurations possess permutation cycles of length 3β consistent with local resonances harbored in isolated hexagons occupied by three particles^12,20^. Finally, permutation cycles of length 4β and 5β only appear in the Z2 phase with, in some cases, 30−40% of particles involved in permutation cycles longer than 1β.”

now reads:

“In the VBS case, 97% of configurations only possess permutation cycles of length 1β. While the remaining 3% of configurations also possess 3β-long permutation cycles. These observations are consistent with the presence of local resonances harbored in isolated hexagons occupied by three particles^12,20^. Finally, permutation cycles of length 4β and 5β only appears in the Z2 phase with, in some cases, 17−20% of particles involved in permutation cycles longer than 1β.”

The original Article has been corrected.

